# The chloride antiporter *CLCN7* is a modifier of lysosome dysfunction in *FIG4* and *VAC14* mutants

**DOI:** 10.1371/journal.pgen.1010800

**Published:** 2023-06-26

**Authors:** Xu Cao, Guy M. Lenk, Vedrana Mikusevic, Joseph A. Mindell, Miriam H. Meisler

**Affiliations:** 1 Department of Human Genetics, University of Michigan, Ann Arbor, Michigan, United States of America; 2 Membrane Transport Biophysics Section, National Institutes of Neurological Disorders and Stroke, Bethesda, Maryland, United States of America; The Jackson Laboratory, UNITED STATES

## Abstract

The phosphatase FIG4 and the scaffold protein VAC14 function in the biosynthesis of PI(3,5)P_2_, a signaling lipid that inhibits the lysosomal chloride transporter ClC-7. Loss-of-function mutations of *FIG4* and *VAC14* reduce PI(3,5)P_2_ and result in lysosomal disorders characterized by accumulation of enlarged lysosomes and neurodegeneration. Similarly, a gain of function mutation of *CLCN7* encoding ClC-7 also results in enlarged lysosomes. We therefore tested the ability of reduced *CLCN7* expression to compensate for loss of *FIG4* or *VAC14*. Knock-out of *CLCN7* corrected lysosomal swelling and partially corrected lysosomal hyperacidification in *FIG4* null cell cultures. Knockout of the related transporter *CLCN6* (ClC-6) in *FIG4* null cells did not affect the lysosome phenotype. In the *Fig4* null mouse, reduction of ClC-7 by expression of the dominant negative *CLCN7* variant p.Gly215Arg improved growth and neurological function and increased lifespan by 20%. These observations demonstrate a role for the *CLCN7* chloride transporter in pathogenesis of *FIG4* and *VAC14* disorders. Reduction of *CLCN7* provides a new target for treatment of *FIG4* and *VAC14* deficiencies that lack specific therapies, such as Charcot-Marie-Tooth Type 4J and Yunis-Varón syndrome.

## Author summary

We previously demonstrated that mutations in the genes *FIG4* and *VAC14* are responsible for human neurological disorders. The pathogenic mechanism of these mutations affects lysosome function, and there are currently no effective treatments available. New therapies for genetic disorders may be developed by identifying modifier genes that interact with the primary causal gene. In the current work, we discovered that *CLCN7* is a modifier of the lysosome dysfunction caused by *FIG4* and *VAC14* mutations. Reducing *CLCN7* expression corrected the structure of lysosomes in cultured cells and was also effective in a mouse model of *FIG4* deficiency. Down-regulation of *CLCN7* thus provides a novel approach to treatment of these severe genetic disorders.

## Introduction

The signaling lipid PI(3,5)P_2_ is generated at the surface of endosomes and lysosomes by a protein complex that includes the kinase PIKFYVE, which synthesizes the lipid, the phosphatase FIG4, which degrades it, and the scaffold protein VAC14 [1–3]. Cells deficient in FIG4 or VAC14 have enlarged lysosomes bounded by membranes containing LAMP1 and LAMP2, and elevated levels of the autophagosome markers LC3-II and p62 [1,4,5]. Surprisingly, since FIG4 is a phosphatase that degrades phosphoinositides, loss of FIG4 leads to decreased levels of PI(3,5)P_2_ in cells [5]. Mutant mice and patients with genetic disorders affecting *FIG4* and *VAC14* develop vacuolization of the CNS [5–8]. The abnormal lysosomes in *FIG4* deficient cells are phase-lucent and distended and appear to be fluid-filled, raising the possibility that they contain elevated solute levels leading to osmotic swelling. If this is the case, elevated hydrostatic pressure and lysosomal membrane tension within the lysosome could impede the recruitment of membrane remodeling components and prevent regeneration of normal lysosomes via tubulation and membrane recycling [9–11].

Mutations of *FIG4* and *VAC14* have been identified in patients with neurodegenerative disorders. The lethal multisystem disorder Yunis-Varón Syndrome results from complete loss-of-function of *FIG4* or *VAC14* [7,12]. Mutations causing partial loss-of-function of *FIG4* are associated with Charcot-Marie-Tooth disease type 4J and familial epilepsy with polymicrogyria [5,13]. Partial loss-of-function mutations of *VAC14* are seen in pediatric-onset striatonigral degeneration and neurodegeneration with impaired CNS myelination [6]. Mice carrying a loss-of-function mutation of *Fig4* display neurological phenotypes and reduced pigmentation [5]. Similar phenotypes, including hypopigmentation and neurological disorders, result from the gain-of-function mutation p.Tyr715Cys in the chloride transporter gene *CLCN7* [14]. The analogous syndromes resulting from disruption of *FIG4* and *CLCN7* raises the possibility of a functional relationship between the two lysosomal proteins they encode. In contrast to the gain-of-function variant p.Tyr715Cys, the impaired trafficking of CLCN7 in the dominant negative variant p.Gly215Arg (mouse p.Gly213Arg) results excess bone deposition and osteopetrosis [15].

PI(3,5)P_2_ directly regulates several lysosomal ion channels and transporters [16–20]. Recently, PI(3,5)P_2_ was demonstrated to influence lysosomal pH via inhibition of the transporter ClC-7 [21]. The CLCN gene family encodes nine CLC transport proteins that function either as Cl^−^ channels or as electrogenic Cl^-^/H^+^ transporters [22–24]. ClC-6 and ClC-7, the proteins encoded by C*LCN6* and *CLCN7*, share 45% amino acid sequence identity and constitute a distinct branch of the CLCN family [25]. The ClC-6 transporter is mainly located in late endosomes, while ClC-7 is located in both lysosomes and late endosomes [24,26,27]. ClC-6 and ClC-7 are Cl^-^/H^+^ exchangers with exchange stoichiometry of 2 Cl^-^ for 1 H^+^ that actively pump Cl^-^ into the endosome and lysosome in exchange for efflux of protons [23,24,28]. ClC-7 has been proposed to be a component of the lysosomal ‘counterion pathway’ which is required for acidification of the organelle, but its role has not yet been fully defined and it may serve to regulate lysosomal [Cl^-^] for other purposes [24,29,30].

The cryo-EM structure of human ClC-7 reveals a positively charged phosphoinositide binding pocket that is conserved in ClC-6 and in the plant anion/proton exchanger atCLC-a [31]. PI(3,5)P_2_ tonically inhibits atCLC-a and human ClC-7 [21,32]. Deficiency of *FIG4* or *VAC14* leads to reduction of PI(3,5)P_2_ [5] that could in turn result in reduced inhibition of ClC-7 and accumulation of Cl^-^. One possible mechanism for the lysosomal swelling could be increased osmotic pressure due to the accumulated Cl^-^, which would lead to osmotic flux into the organelle. This model would connect the dysregulation of H^+^/Cl^-^ antiport to the vacuole formation observed in FIG4 and VAC14 deficiency or ClC-7 gain-of-function.

To probe the connections between alterations in the PI(3,5)P_2_ pathway and lysosomal chloride/proton exchange, we examined gene interaction between *FIG4*, *VAC14*, *CLCN6* and *CLCN7* by pairwise combination of mutant genes in cultured cells and mutant mice. The results support a role for *CLCN7* in the lysosome dysfunction of *FIG4* and *VAC14* mutants, represented in the final figure, and suggest that inhibitors of this chloride transporter may be therapeutic for *FIG4* and *VAC14* deficiency disorders.

## Methods

### Ethics statement

All experiments described involving animals were in accordance with institutional animal care and use guidelines, and experimental protocols were approved by the Institutional Animal Care and Use Committee (IACUC) at the University of Michigan under animal protocol number PRO00009797.

### Cell culture and imaging

Human HAP1 cells (Horizon Discovery, #C631) were maintained in Iscove’s Modified Dulbecco’s Medium (IMDM) supplemented with 10% FBS, 100 units/mL penicillin, and 100 μg/mL streptomycin (Invitrogen). Cells were cultured in a humidified incubator at 37 °C with 5% CO2. Transfection of plasmids into HAP1 cells was carried out with Lipofectamine 3000 (Life Technologies) following manufacturer’s instructions. Cell morphology was evaluated with phase contrast images taken at 20x with the EVOS FLc system (Life Technologies). Visible vacuoles (>0.4 um) were measured using ImageJ software [33]. Ratiometric measurement of lysosomal pH was carried with the dye Oregon Green 488-dextran as previously described [21]. LAMP2 antibody # H4B4 was obtained from the Developmental Studies Hybridoma Bank, University of Iowa, and used at 3 ug/ml. The secondary antibody was anti-Mouse IgG Alexa fluor plus 488 from Invitrogen, A32723, used at 1/2000 dilution.

### Generation of CRISPR edited HAP1 cell lines

Guide sequences targeting exon 2 of the *FIG4* gene were designed with E-CRISP software [34] and obtained from IDT (Integrated DNA Technologies). The guide oligos were cloned into plasmid pSpCas9 (BB)-2A-Puro (PX459) V2.0 (Addgene # 62988) for expression of the sgRNA and Streptomyces pyrogenes Cas9. After 48 hours of selection for puromycin resistance, individual HAP1 cells were isolated by flow sorting into 96 well plates. FIG4 null clones were visually identified by their content of large vacuoles. Exon 2 of *FIG4* was amplified from vacuolated clones and indels were identified by Sanger sequencing. The *VAC14* null HAP1 cell line G9 was generated previously using the same method [35]. Guide RNAs targeting *CLCN6* and *CLCN7* were selected from the GeCKO library [36]. sgRNAs were obtained from IDT and cloned into pSpCas9 (BB)-2A-Puro (PX459) V2.0 as above. After transfection and puromycin selection, single cells were isolated by flow-sorting, plated in 96 well plates, and grown to confluence. Targeted exons were isolated with the TOPO TA cloning kit from Invitrogen (Cat. # 45–0071) and examined by Sanger sequencing to identify indels and in-frame termination codons. Wildtype transcripts were not detected in the selected null clones for *FIG4*, *VAC14*, *CLCN6* and *CLCN7*.

### FACS analysis of lysosome enlargement

Fluorescence-activated cell sorting (FACS) was carried out as previously described [35]. HAP1 cells were cultured in 6-well plates for 18 hours and labeled for 15 min with 5 μM LysoSensor Yellow/Blue DND-160 dye (PDMPO) (Invitrogen #L7545). After washing three times with PBS, cells were removed from plates by treatment with TrypLE Express Enzyme (Thermo Fisher Cat #12604013), suspended in PBS containing 2% FBS, and transferred to 5 mL flow vials on ice. The fluorescence of LysoSensor DND-160 was recorded in the yellow spectrum to detect variation in lysosome content, with excitation at 329 nm and emission at 546 nm. Cells were sorted in a MoFlo Astrios Cell Sorter (Beckman Coulter, Inc) in the University of Michigan Flow Cytometry Core.

### Animals

The *Fig4* null mouse mutant, also designated pale tremor (*plt*), arose spontaneously by transposon insertion on a mixed strain background. Two congenic mouse *Fig4* null lines were subsequently generated, by >30 generations of backcrossing to strain C57BL/6J and strain C3H/HeJ [37]. *Clcn7-G213R* mice were generated by targeting 129Sv ES cells [15] and kindly provided by Dr. Michael J Econs, Indiana University School of Medicine. The current *Clcn7-G213R* line was confirmed to be congenic on strain C57BL/6J by SNP genotyping with the miniMUGA panel (Neogen). To measure hindlimb clasping, mice were suspended for 1 min and the % time with hindlimbs retracted was measured [38]. For the hanging wire test, mice were placed on the wire mesh cage lid, the lid was inverted, and the time to falling was measured. The human CLCN7 variant p.Gly215Arg corresponds to p.Gly213Arg in the mouse gene. Histology on H&E stained sections of brain and dorsal root ganglia was carried out at Histoserv (Bethesda, MD). The percent of the deep cerebellar nucleus occupied by vacuoles was determined using ImageJ and Adobe Photoshop. Mice in this study were housed and cared for in accordance with NIH guidelines in a 12/12h light/dark cycle with standard mouse chow and water available *ad libitum*.

## Results

### Generation of a *FIG4* null HAP1 cell line

**Fig 1:**
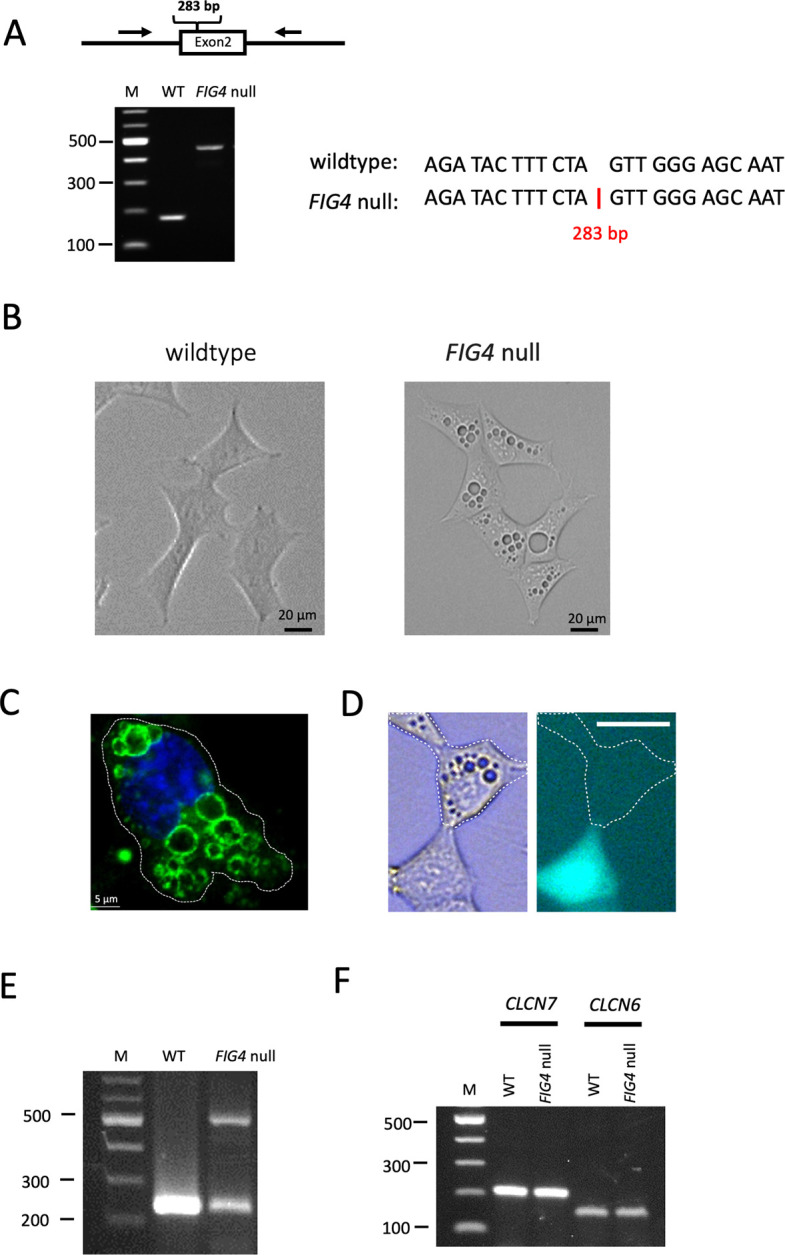
Generation of *FIG4* null HAP1 clone F. Human HAP1 cells were transfected with Cas9 and an sgRNA complementary to exon 2 of *FIG4* (S1 Fig). Transfected cells were cloned and screened visually for enlarged vacuoles. Line F was evaluated by Sanger sequencing. (A) Gel electrophoresis of PCR products containing exon 2 from wildtype and mutant cells. The mutant line contains a 283 bp insert at the indicated position in exon 2. The sequence of the insert is shown in S1 Fig. (B) Vacuoles in *FIG4* null line F that are not present in wildtype HAP1 cells. (C) The vacuoles are positive for LAMP2, indicating that they are enlarged lysosomes. (D) Transfection of wildtype FIG4 cDNA corrects vacuolization. Transfected cells are identified by fluorescence of the co-transfected GFP. Scale bar, 25 um. (E) Demonstration of expression of CLCN7 and CLCN6 in wildtype and FIG4 null HAP1cells by RT-PCR.

The *FIG4* null cell line was generated by transfection of HAP1 cells with Cas9 and an sgRNA complementary to exon 2 (Fig 1). Individual mutated cells were sorted into 96 well plates and subjected to clonal expansion. During culture, the haploid HAP1 cells reverted to the more stable diploid state [39]. Sequencing of vacuolated clones identified the *FIG4* null cell line (FIG4-F). The *FIG4* gene contains a 283 bp insertion downstream of c.78A that results in the protein truncation allele p.Leu26_*_8 (Figs 1A and S1). The *FIG4* null cells are filled with large spherical vacuoles (Fig 1B), bounded by a membrane that is positive for the lysosomal membrane marker LAMP2 (Fig 1C), consistent with the previously described *FIG4* null phenotype [4,5]. Transfection with wildtype *FIG4* cDNA corrected vacuolization (Fig 1D). To confirm the lack of wildtype *FIG4* expression, we amplified exon 2 by RTPCR. Two products were obtained, with lengths of 200 and 500 bp (Fig 1E). TA cloning and Sanger sequencing demonstrated that the 500 bp RTPCR product contains the entire 283 bp insert with an in-frame stop codon encoding the protein Leu26*8 (S1 Fig). The 200 bp RTPCR product is spliced from exon 1 to a splice acceptor site near the 3’ end of the 283 bp insert, and encode the protein product Arg21*1(S1 Fig). There was no expression of wildtype *FIG4* transcript in the HAP1 null line.

The expression of *CLCN6* and *CLCN7* in wildtype HAP1 cells and FIG4-F null cells was also demonstrated by RT-PCR (Fig 1F).

### Correction of enlarged lysosomes in *FIG4* null cells by knockout of *CLCN7*

**Fig 2:**
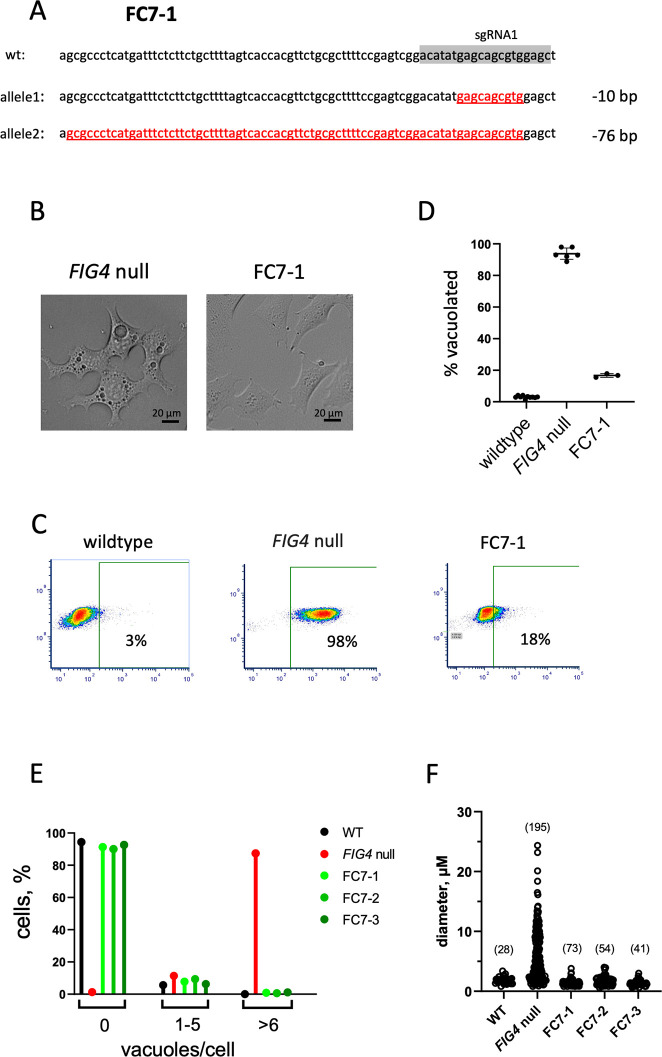
Knockout of *CLCN7* in *FIG4* null cells rescues vacuolization. (A) The *FIG4* null cell line F was transfected with Cas9 and an sgRNA complementary to exon 11 of *CLCN7* (shown in grey). Transfected cells were cloned and *CLCN7* exon 11 was sequenced. Line FC7-1 contains allelic exon 11 deletions of 10 bp and 76 bp (red). (B) Vacuoles in *FIG4* null cells are corrected by knockout of *CLCN7* in line FC7-1. (C). Quantitation of cell vacuolization by FACS. Cells are incubated with Lysosensor DND160 for 15 min and washed 3x with PBS. Cells with enlarged vacuoles retain elevated content of the fluorescent dye. With the indicated gating, 97% of FIG4 null cells contain elevated fluorescence; this is reduced to 18% in cell line FCL7-1 by knockout of *CLCN7*. (D) Quantitation of % cells with vacuoles in replicate FACs experiments. (E). Count of vacuoles per cell in WT (n = 376 cells), FIG4 null (n = 395), and three double-knockout cells, FC7-1 (n = 334), FC7-2 (n = 341) and FC7-3 (n = 366). (F). Size of vacuoles in the 5 cell lines shown in panel E. The number of vacuoles measured is indicated.

To evaluate the role of CLCN7 in *FIG4*-related lysosome dysfunction, we generated double knockout cells. The *FIG4* null cells were transfected with a plasmid expressing Cas9 and three different sgRNAs targeting CLCN7. The sgRNA sequences are shown in Fig 2A (highlighted in grey) and S2 Fig. Three subclones designated FC7-1, FC7-2 and FC7-3 were isolated, one from each sgRNA. The *FIG4* null cells reverted to the stable diploid state [39] and Sanger sequencing demonstrated that each *CLCN7* null cell line contains two allelic indels (Figs 2A and S2). These six indels in *CLCN7* all generate null alleles with in-frame stop codons leading to premature protein truncation.

In contrast to the parental *FIG4* null line, all three subclones that are also null for *CLCN7* exhibit normal morphology and lack enlarged lysosomes (Figs 2B and S2). The effect of inactivation of *CLCN7* on correcting lysosome morphology was quantitated by FACs sorting.

Line FC7-1 was incubated with fluorescent Lysosensor DND160 and the distribution of cell fluorescence was measured. The proportion of cells with elevated lysosomal fluorescence in the *Fig4* null cells was 94 ± 4% (mean ± SD, n = 3) and was dramatically reduced in the double mutant lines (e.g FC7-1, Fig 2C, p< 0.0001, two tailed t-test). The percent of cells without vacuoles was restored to wildtype level in all 3 double mutant lines (Fig 2E). The diameter of the enlarged vacuoles in *FIG4* null cells, which varied up to 30 uM, was restored to normal in the double mutant cells (Fig 2F). The striking correction of vacuolization by knockout of *CLCN7* suggests a key role for ClC-7 in the enlarged lysosomes of *FIG4* null cells.

### Lysosomal pH in *FIG4*^*-/-*^,*CLCN7*^*-/-*^ double knockout cells

**Fig 3:**
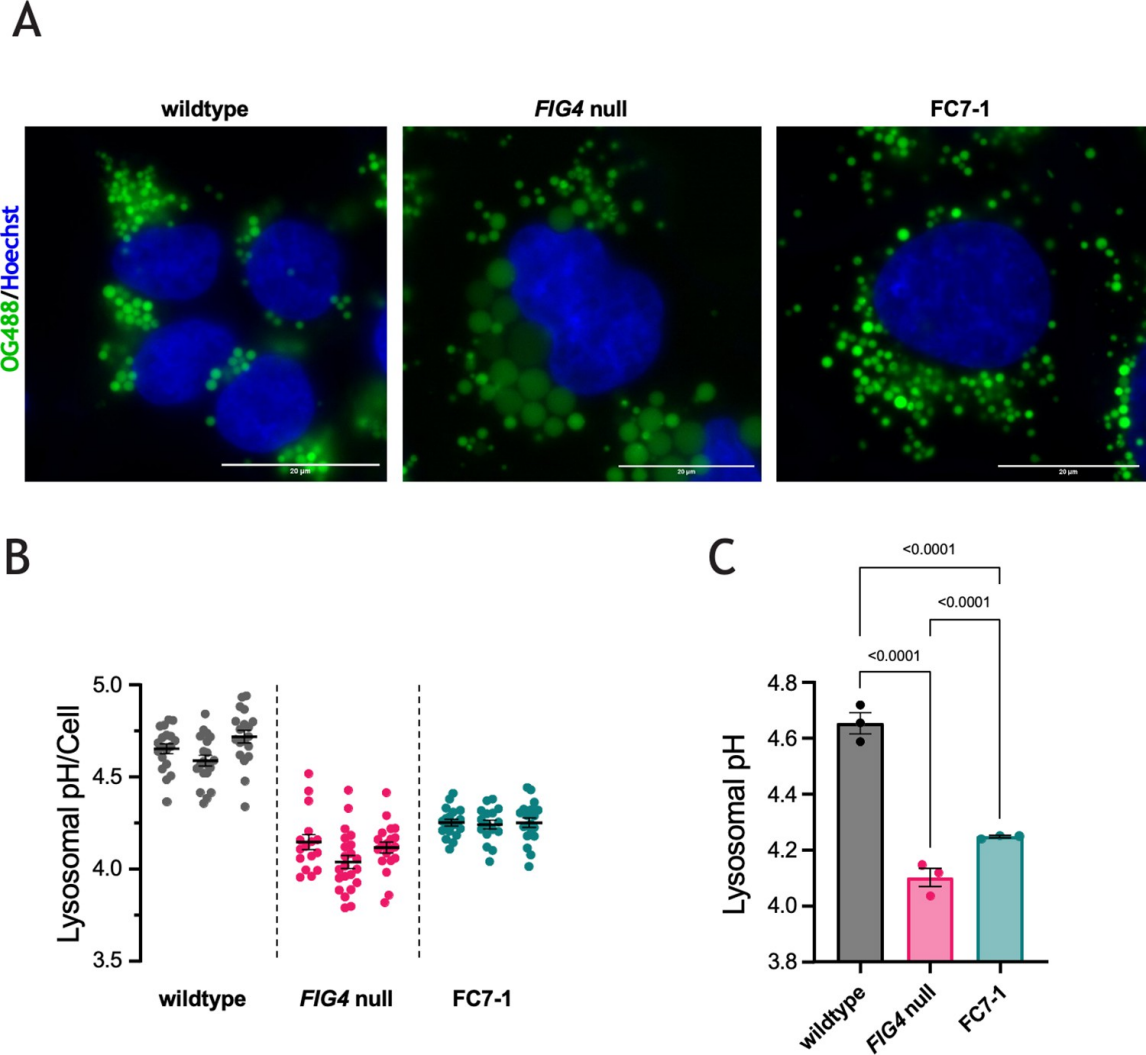
Knockout of CLCN7 reduces lysosomal hyperacidity in *FIG4* null cells. Lysosomal pH was measured with a ratiometric assay [21]. The pH of lysosomes in *FIG4* null cells is hyperacidic compared with wildtype cells [35]. (A) Representative live cell images of HAP1 wildtype, *FIG4* null, and FC7-1 cells stained with Oregon Green 488-dextran (OG488) (green) and Hoechst 33342 (blue). Scale bar, 20 μm. (B) The low lysosomal pH in *FIG4* null cells is partially corrected by knockout of *CLCN7* in FC7-1 cells. Each symbol represents the average pH of lysosomes in one cell. Data from three replicate experiments are shown. (C) Lysosomal pH is increased from pH 4.1 in *FIG4* null cells to pH 4.25 in FC7-1 cells, which represents an 0.7-fold decrease in proton concentration in the lysosomal lumen. Each symbol represents the average lysosomal pH from one experiment in panel B. In panels B and C, mean values are indicated with error bars indicating Standard Error of the Mean. P-values from 2-way ANOVA.

**Fig 4:**
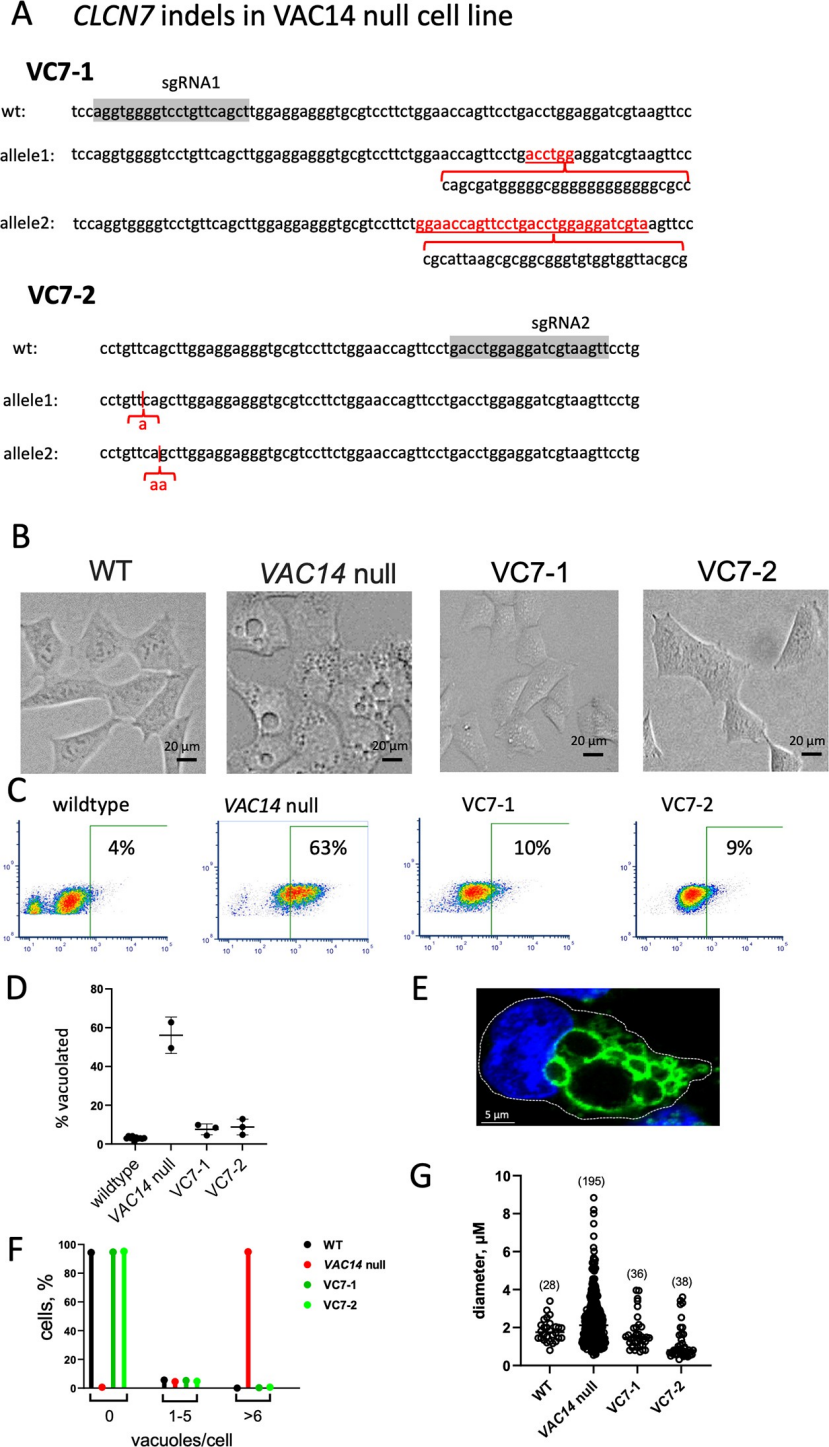
Knockout of *CLCN7* in *VAC14* null cells corrects vacuolization. Lysosomal enlargement in the VAC14 null HAP1 line G9 was previously described [35]. (A). Exon 11 of *CLCN7* was targeted in line G9 by transfection of Cas9 with the indicated sgRNAs, and cloned cell lines were sequenced. Line VC7-1 contains allelic deletions (underlined, red) and inserted sequences (black). (B) Knockout of *CLCN7* in lines VC7-1 and VC7-2 corrects the vacuolization of *VAC14* null cells. (C) Quantitation of % vacuolated cells by FACs, with the indicated proportion of vacuolated cells identified by elevated fluorescence. (D) Replicate FACs sorting experiments. The % vacuolated cells is reduced from 63% in VAC14 null line G9 to 9% in double knockout lines VC7-1 and VC7-2 (p <0.01, two-tailed t-test). (E) Vacuoles in VAC14 knockout cells stained for the lysosomal membrane marker LAMP2. (F) Number of vacuoles per cell in WT HAP1 cells (n = 376), VAC14 null cells (n = 330), and double mutant lines VC7-1 (n = 301) and VC7-2 (n = 314). (G) Sizes of vacuoles in the 4 cell lines in panel F.

We previously reported that the pH of the enlarged lysosomes in *FIG4* null HAP1 cells is significantly more acidic than in wildtype cells [35]. Knockout of *CLCN7* in wildtype cells does not affect lysosomal pH [21,40,41]. To determine the impact of knockout of *CLCN7* in the *FIG4* null cells, we loaded lysosomes with Oregon Green 488-dextran and quantified lysosomal pH by ratiometric analysis. Calibration curves are shown in S3 Fig. In contrast to cells from patients with gain of function variants of *CLCN7* [14], in the *FIG4* null cells the extensive overlap between compartments staining with Oregon Green 488-dextran (OG488) and Lysotracker Blue indicates that the dextran-coupled dye is efficiently delivered to the large vacuoles (S4 Fig). The average lysosomal pH of the *FIG4* null line was pH 4.10, compared with pH 4.65 in wildtype cells (Fig 3). Knockout of *CLCN7* in the *FIG4* null cells resulted in an increase of 0.15 pH units (Fig 3), equivalent to a ~29% decrease in proton concentration in the lysosomal lumen. This partial correction in the double mutant line indicates that *CLCN7* contributes to hyperacidity in *FIG4* null cells, in combination with additional mechanisms that may play a role.

### Vacuolization *of VAC14* null cells is also rescued by knockout of *CLCN7*

VAC14 is a scaffold protein with repeated HEAT domains that is co-localized with FIG4 in the PI(3,5)P_2_ biosynthetic complex. Like *FIG4*, loss of *VAC14* results in reduced PI(3,5)P_2_ and enlarged lysosomes [1]. To determine whether inactivation of *CLCN7* is protective against loss of *VAC14*, the HAP1 null cell line G9 [35] was transfected with Cas9 and two different sgRNAs targeting exon 11. Two clonal lines containing biallelic null mutations in exon 11 of CLCN7 were isolated and designated VC7-1 and VC7-2 (Fig 4A). The enlarged lysosomes of the *VAC14* null cells were corrected in both double knockout lines (Fig 4B). The 43% of cells with enlarged lysosomes in the VAC14 null line was reduced to 9% and 10% in double knockout lines VC7-1 and VC7-2 (Fig 4C and 4D). LAMP2 staining of the vacuoles in VAC14 null cells is shown in Fig 4E. The number of vacuoles per cell and the size of vacuoles were also returned to normal by inactivation of *CLCN7* (Fig 4F and 4G). This result is consistent with the proposed role of *CLCN7* in the response to the low PI(3,5)P_2_ levels in *VAC14* mutant cells [1].

### Inactivation of *CLCN6* does not rescue *FIG4* null cells

**Fig 5:**
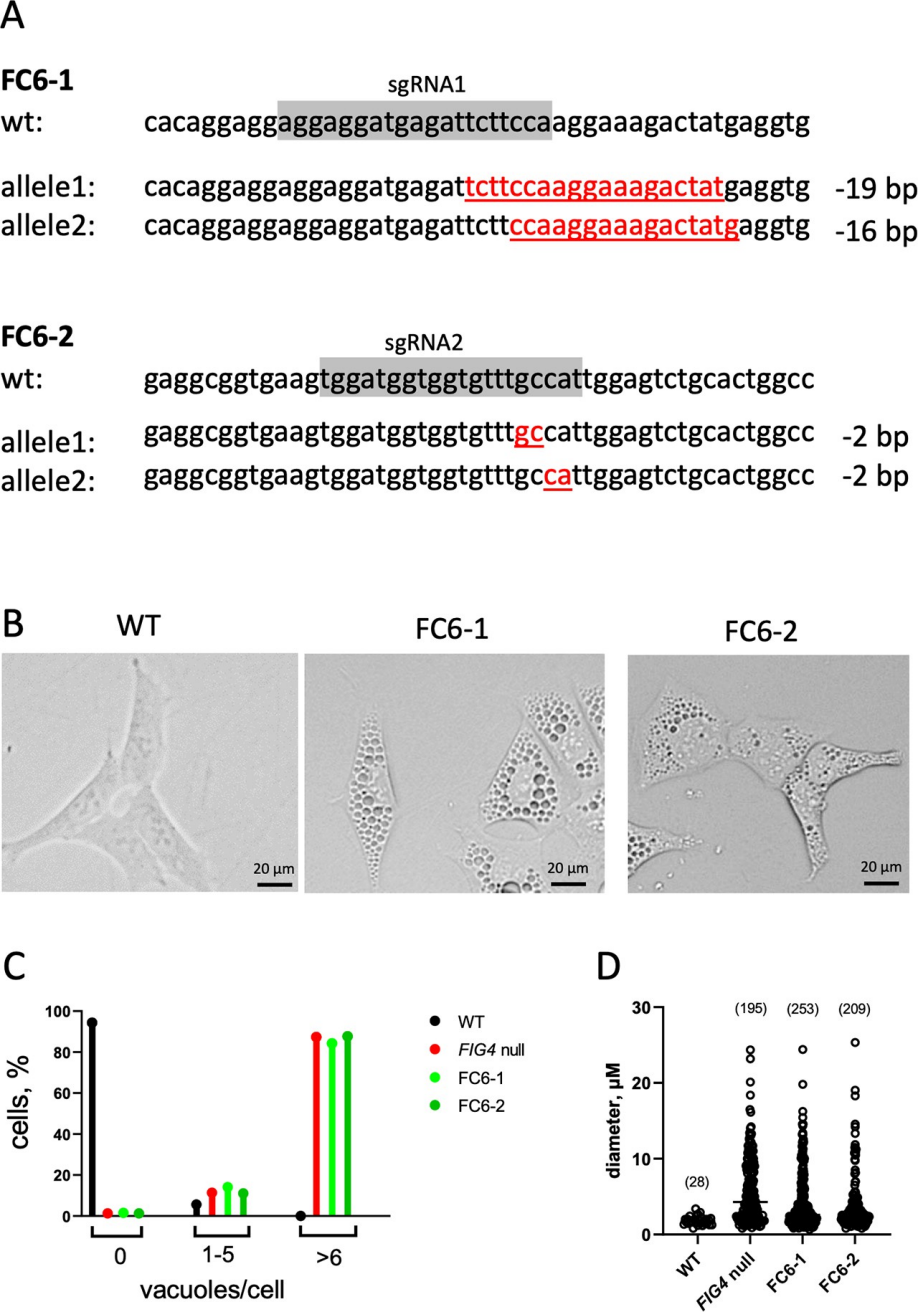
Knockout of *CLCN6* does not rescue enlarged lysosomes in *FIG4* null cells. Chloride transporter gene *CLCN6* was targeted in the *FIG4* null HAP1 cell line as described in the text. (A) Targeted deletions of *CLCN6* in exon 2 (line FC6-1) and exon 6 (line FC6-2). Both lines are compound heterozygous for allelic null mutations. (B) The vacuolated phenotype of the FIG4 null line (Fig 1) was not corrected by knockout of *CLCN6* in lines FC6-1 and FC6-2. (C) Percent of cells with the indicated number of vacuoles in wildtype HAP1 (n = 376), FIG4 null (n = 375), FC6-1 (n = 318) and FC6-2 (n = 317). (D) Size of vacuoles in the 4 cell lines from panel C. The number of vacuoles measured is indicated.

The amino acid sequences of the ClC-6 and ClC-7 transporters are 55% divergent, and the two proteins were reported to differ in subcellular localization, with *CLCN6* restricted to the endosome [26]. To determine whether knockout of *CLCN6* also alters the enlarged vacuole phenotype of *FIG4* null cells, we transfected the *FIG4* null cell line with Cas9 and sgRNAs targeting exon 2 and exon 4 of *CLCN6*. Two double mutant cell lines with biallelic frame-shifting indels in *CLCN6* were isolated (Fig 5A). Both double mutant cell lines were as highly vacuolated as the *FIG4* null parent line (Fig 5B and 5C). The size of the vacuoles in these double mutant lines (Fig 5D) did not differ from the *FIG4* null lines (Fig 2F). The failure of *CLCN6* disruption to correct vacuolation of *FIG4* null cells demonstrates divergence in the function of these two members of the *CLCN* gene family.

### In *Fig4* null mice, reduction of *Clcn7* ameliorates mutant phenotypes

**Fig 6:**
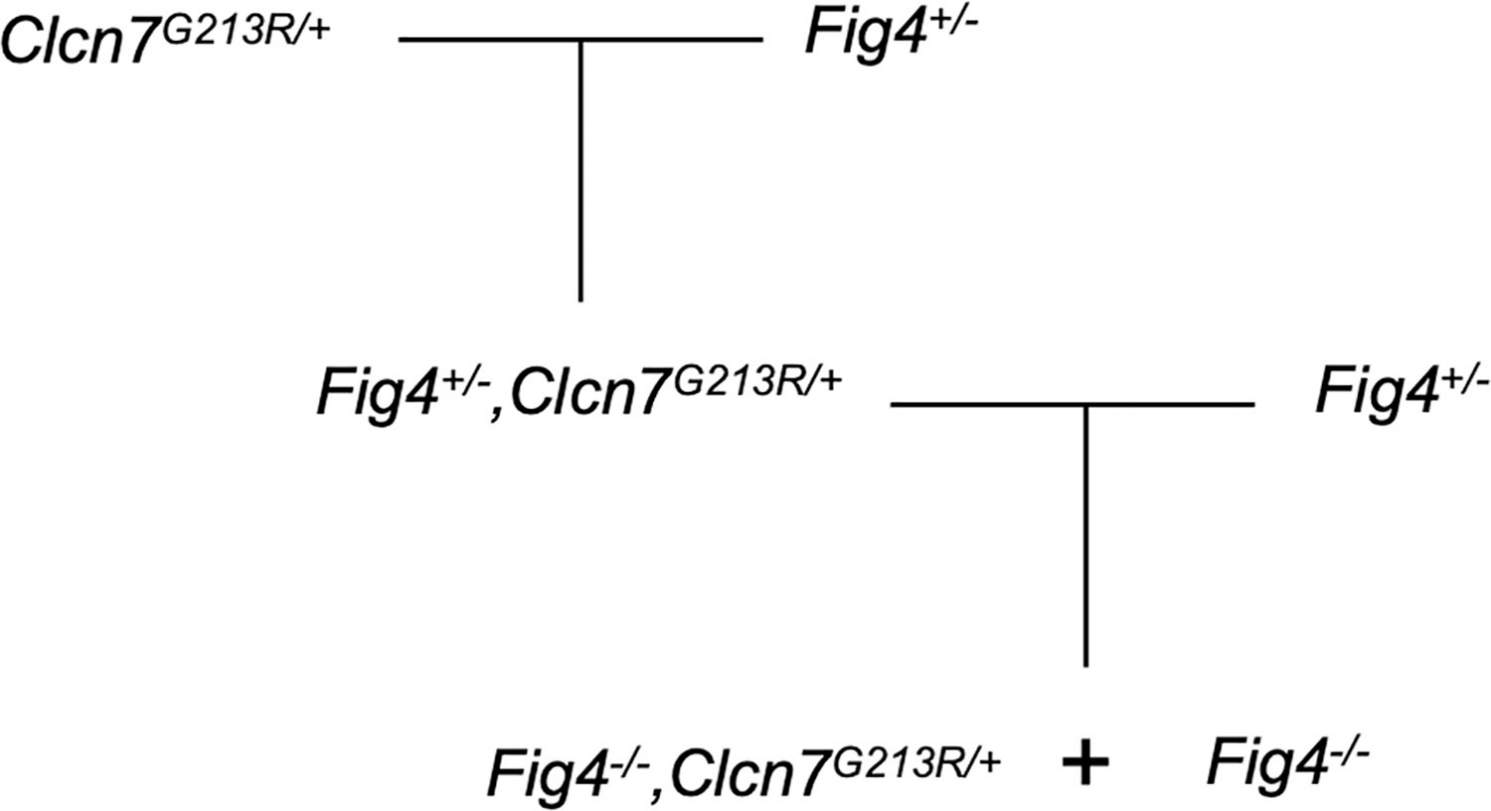
Breeding scheme to generate double mutant mice that are null for *Fig4* and carry the dominant negative mutation of *Clcn7*. Heterozygous *Clcn7*^*G213R/+*^ mice carrying a dominant negative patient mutation were crossed with heterozygous *Fig4* null mice. The double heterozygous offspring were crossed with *Fig4* heterozygous null mice to generate homozygous *Fig4* null mice with the *Clcn7* mutation and control *Fig4* null mice without the *Clcn7* mutation.

To determine the impact of knockdown of *CLCN7* on phenotypes caused by low PI(3,5)P_2_ in a whole animal model, we used *Fig4* null mice [5]. To reduce *Clcn7* function, we crossed *Fig4* heterozygous null mice with a mutant carrying the dominant negative human mutation *CLCN7*-p.Gly215Arg. This mutation causes a trafficking defect with a dominant negative effect on the dimeric ClC-7 transporter [42]. Heterozygous *Clcn7*^*G213R/+*^ mice on the C57BL/6J strain background are viable with mild osteopetrosis [15]. We crossed male *Clcn7*^*G213R/+*^ mice with heterozygous female *Fig4*^*+/-*^ mice, also on strain C57BL/6J, to generate *Fig4*^*+/-*^,*Clcn7*^*G213R/+*^ double heterozygotes. Subsequent crossing with *Fig4*^*+/-*^ mice on the C3H/HeJ strain background generated (C3H/HeJ × C57BL/6J) F1 mice that were null for *Fig4* and also carried the *Clcn7*^*G213R*^ allele (Fig 6).

**Fig 7:**
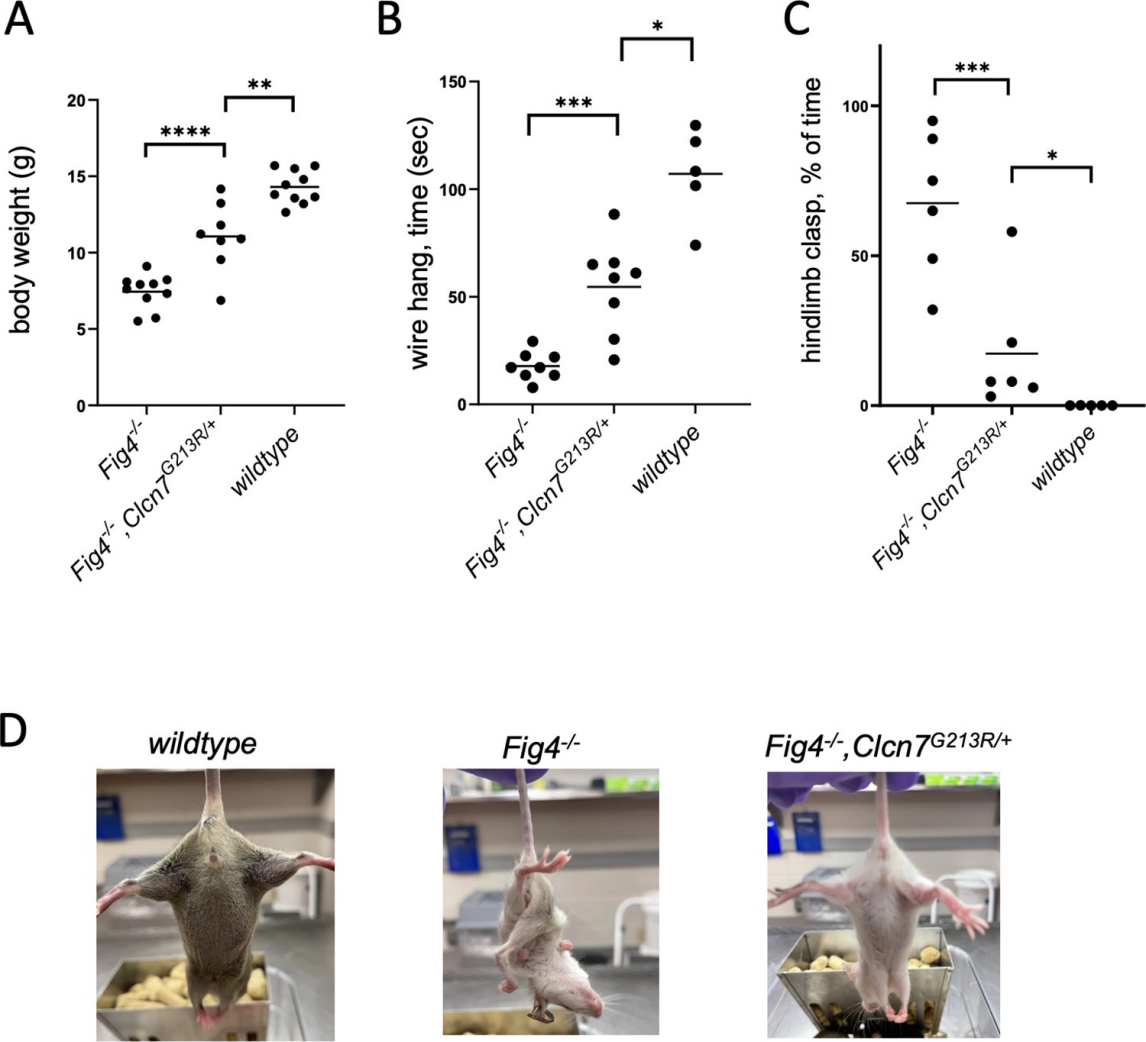
Improved phenotypes of *Fig4* null mice carrying a dominant negative mutation of *Clcn7*. (A) The body weight of *Fig4* null mice at P21 is approximately half the weight of wildtype littermates. The *Clcn7* mutation provided partial correction of the growth deficit. Data from male and female mice, which do not differ at P21, and from multiple litters all reduced to include 5 offspring at P5. (B) At postnatal day P30, *Fig4* null mice are unable to support their weight in the wire hang test. Addition of the *Clcn7* mutation improved performance. (C, D) Wildtype mice splay their hindlimbs in the tail suspension test while neurological mutants clasp their hindlimbs. Mice were tested at postnatal day 30 with a 1 minute test time. The extreme clasping of hindlimbs and forelimbs in *Fig4* null mice is maintained for more than 50% of the test time. Addition of the *Clcn7* mutation resulted in a less severe clasping for 10% of test time.

On the (C57BL/6J x C3H)F1 strain background, *Fig4* null mice exhibit diluted pigmentation, small size, vacuolization and degeneration of the CNS and PNS, and survival for 4 to 6 weeks [5,37]. The presence of the *Clcn7* mutation in *Fig4*^*-/-*^,*Clcn7*^*G213R/+*^ mice resulted in increase of body weight at P21 from 7.5 ± 0.4 grams to 11.1 ± 0.8 grams (p<0.0005) (Fig 7A). There was also improved performance on two tests of neuromuscular function. On postnatal day 30, *Fig4* null mice could support their weight in the hanging wire test for only 18 ± 2 seconds before falling, while *Fig4*^*-/-*^,*Clcn7*^*G213R/+*^ mice remained for 55 ± 8 seconds (Fig 7B). Abnormal hindlimb and forelimb clasping persisted for > 50% of test time in *Fig4* null mice (Fig 7C). In *Fig4*^*-/-*^,*Clcn7*^*G213R/+*^ mice, limb clasping was less extreme and persisted for only 17% of test time (Fig 7C).

**Fig 8:**
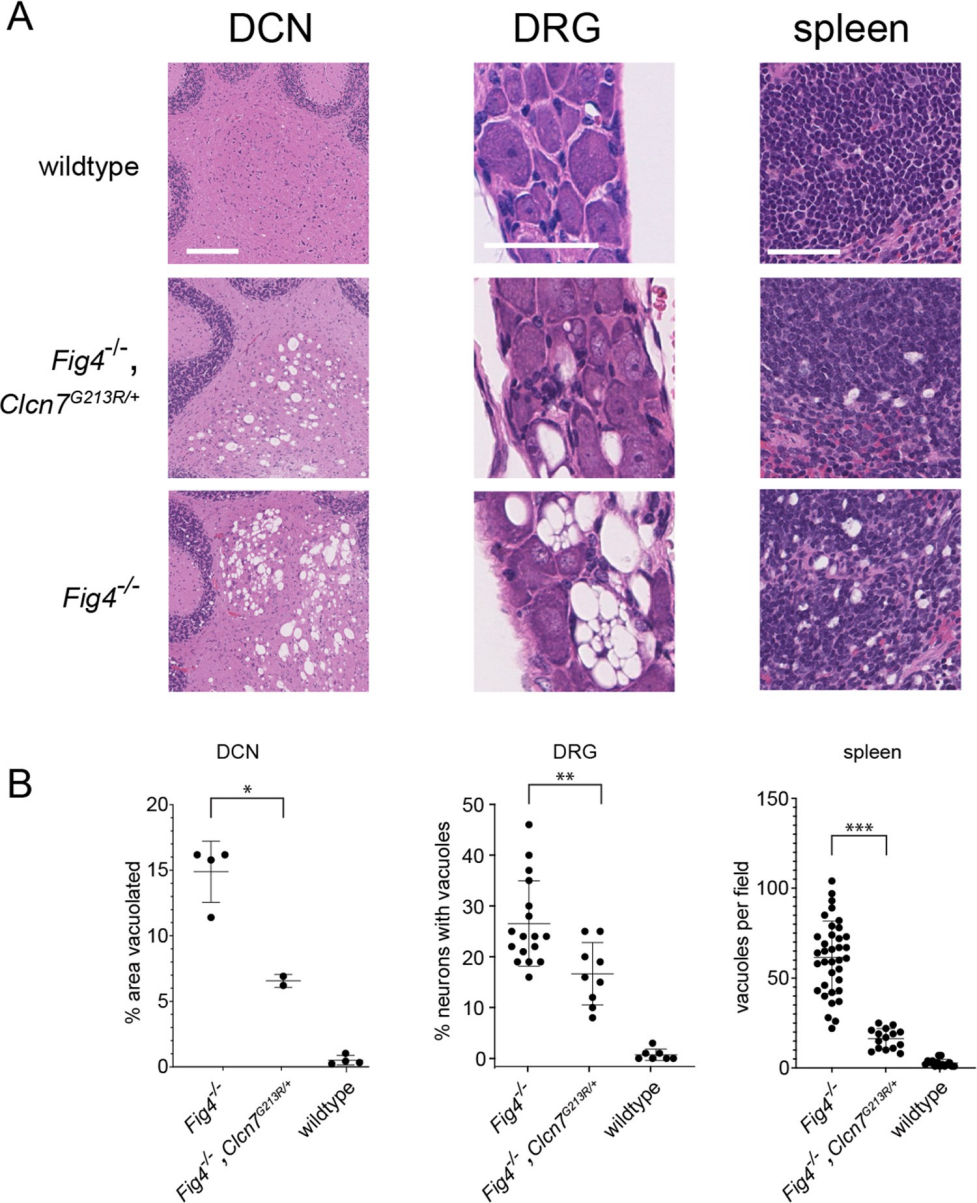
Partial *in vivo* correction of cell vacuolization in *Fig4*^*-/-*^,*Clcn7*^*G213R/+*^ mice. Tissues from 3 week old mice were stained with haematoxylin and eosin. A. Representative images. DCN: deep cerebellar nuclei; DRG: dorsal root ganglia; spleen. B. Quantitation. **DCN**, each symbol represents the DCN from a single sagittal section. Vacuoles accounted for the indicated % of the area of the DCN. **DRG**: Each symbol represents one section of an entire DRG containing approximately 100 neuronal soma. The percent of neuronal soma containing vacuoles was counted. **Spleen**: Each symbol represents one field. The number of vacuoles per field was counted. Scale bars, DCN 200 um; DGR 50 um; Spleen, 50 um. Statistical comparison of mean ± std. dev. was determined by unpaired t-tests. * p<0.01; ** p<0.005; *** p<0.0001.

Histological examination of tissues from the *Fig4*^*-/-*^,*Clcn7*^*G213R/+*^ double mutant mice revealed partial rescue of tissues affected in the *Fig4* null plt mice. The earliest vacuolization in the *plt* mice is seen in the deep cerebellar nucleus at E16 [5]. In the double mutant mice, the deep cerebellar nucleus at 30 days of age is partially corrected, compared with *Fig4* null mice (Fig 8A and 8B). Dorsal root ganglia are extensively vacuolated in the *plt* mutant, and partially protected in the double mutant mice (Fig 8A and 8B). Spleen is also highly vacuolated in *Fig4* null mice, and partially restored in the double mutant mice (Fig 8A and 8B).

**Fig 9:**
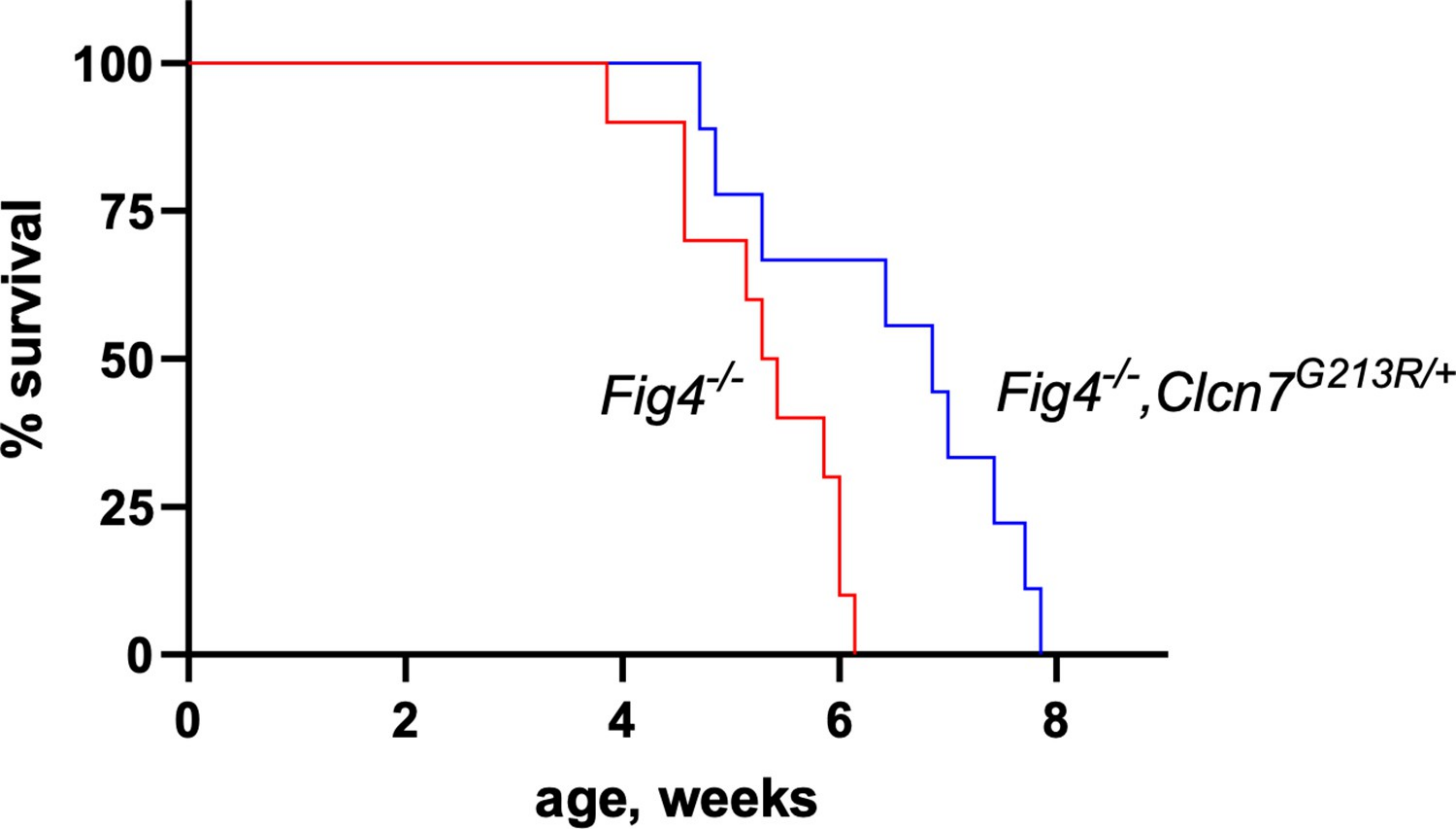
Extended survival of *Fig4* null mice with the *Clcn7* mutation. The mean survival of *Fig4* ^-/-^ mice (38 days). was increased to 48 days in *Fig4*^*-/-*^, *Clcn7*^*G213R/+*^ mice (p = 0.009, Log-rank (Mantel-Cox) test). Maximal survival increased from 6 weeks to 8 weeks.

Consistent with the improved behavior and pathology, maximal survival of mutant mice was increased from 6 weeks for *Fig4* null mice to 8 weeks in *Fig4*^*-/-*^,*Clcn7*^*G213R/+*^ mice (p<0.01, Log Rank Test) (Fig 9).

In summary, the improved lysosome morphology brought about by knockout of *CLCN7* in cultured *FIG4* null HAP1cells was confirmed *in vivo* in the double knockout mice by improved histopathology and extended survival. The data suggest that reduction of *CLCN7* activity could positively impact human genetic deficiencies human *FIG4* and *VAC14*.

## Discussion

PI(3,5)P_2_ is emerging as an important regulator of lysosomal physiology and ion homeostasis. Previous work demonstrated that depletion of PI(3,5)P_2_ by PIKFYVE inhibition or by FIG4 mutation results in altered lysosomal membrane dynamics. PI(3,5)P_2_ modulates a number of known lysosomal and endosomal ion channels and transporters, including TRPML1 [43] and TPC1 [16,17,19,37,44,45], though the physiological significance of this modulation is unclear. More recently, PI(3,5)P_2_ was shown to inhibit ClC-7 function, thereby limiting the normal extent of acidification by the v-type ATPase [21]. Coordinated regulation of multiple channels and transporters may be important for simultaneously controlling lysosomal pH, ion composition, and osmotic balance.

Inactivation of *FIG4* or *VAC14* in HAP1 cells results in accumulation of enlarged hyperacidic vacuoles derived from lysosomes [35]. Because of the similarity between these and the vacuoles formed upon ClC-7 gain-of function, we examined the role of *CLCN7* in this process by generating cell lines with mutations of the phosphoinositide biosynthetic genes *FIG4* or *VAC14* together with mutations of the lysosomal chloride transporter genes. Remarkably, knockout of *CLCN7* reduced vacuole formation in *FIG4* null and *VAC14* null cells. Knockout of *CLCN7* also increased vacuolar pH in *Fig4* null cells (though not to normal levels), supporting the role of ClC-7 in vacuolar acidification of PI(3,5)P_2_ deficient cells [21,35]. These observations suggest that the chloride ions carried by ClC-7 and/or the resulting pH changes are essential effectors of the lysosomal swelling in PI(3,5)P_2_ deficiency. Interestingly, the lysosomal swelling is corrected by the lysosomotrophic agent chloroquine, which also alkalinizes lysosomes in cultured cells [14,46].

Gain-of-function mutations of *CLCN6* and *CLCN7* induce enlarged lysosomes in mammalian cells similar to those of PI(3,5)P_2_ deficient cells, raising the possibility that accumulation of Cl^−^ can induce lysosome enlargement [14,47]. While knockout of *CLCN7* corrected the enlarged lysosomes in *FIG4* null cells, knockout of the paralog *CLCN6* did not, demonstrating a difference between the two evolutionarily related transporters. This may result from a difference in subcellular, recently described functional differences [48], or lack of CLCN6 protein expression in HAP1 cells. Although expression of the *CLCN6* transcript was detected in HAP1 cells, and only mutant transcript was found in mutant *CLCN6* mutant cells, we were unable to confirm translation of this transcript in wildtype HAP1 cells. Since expression of *CLCN6* protein may be limited to neurons [26], the difference between CLCN7 and CLCN6 in our experiments may reflect a difference in expression of the protein rather than a difference in their role in response to phosphoinositides. Other evidence suggests that lysosomal enlargement and hyperacidification due to PI(3,5)P_2_ depletion might occur through divergent mechanisms, suggesting a more complex mechanism of lysosomal size change [21].

**Fig 10:**
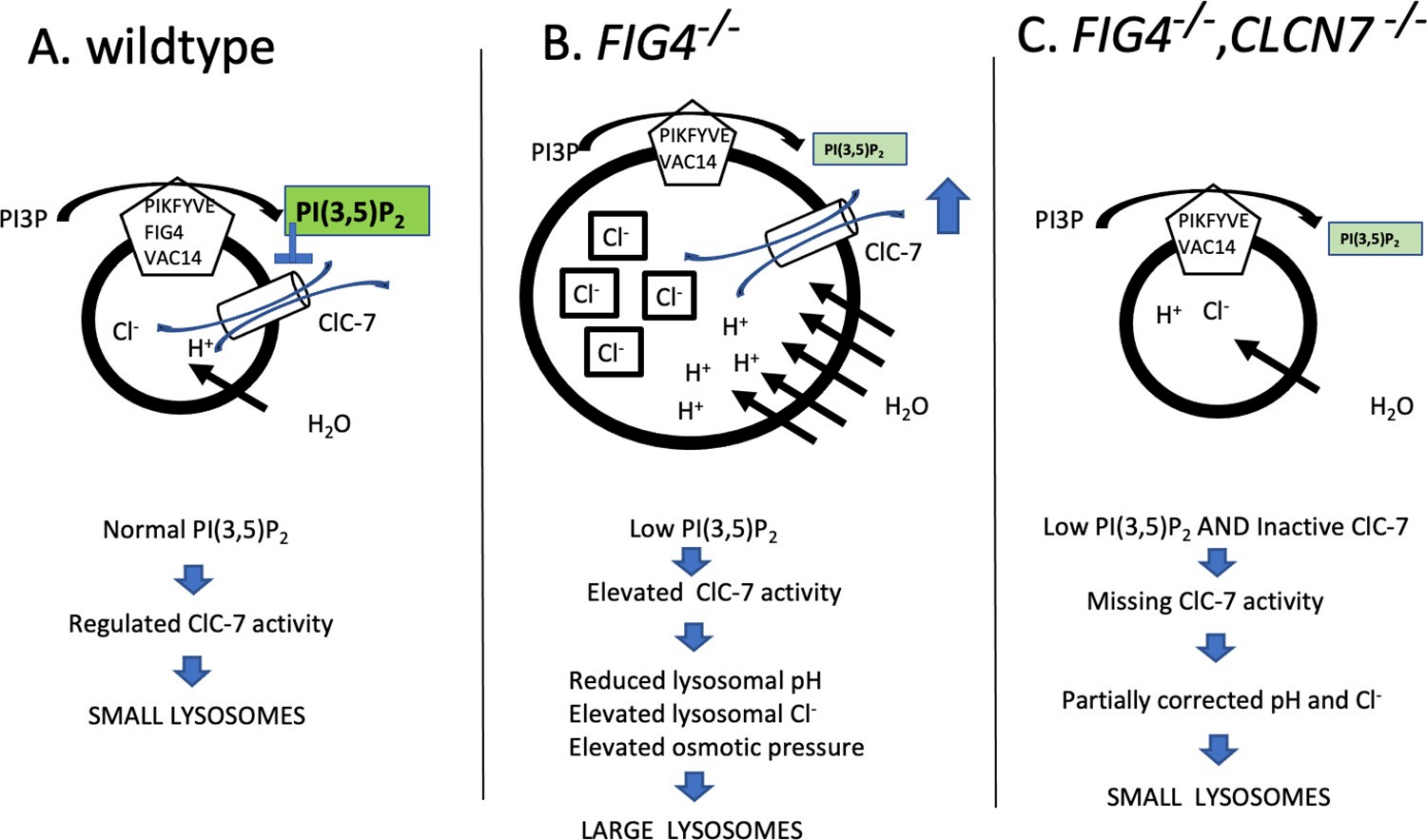
Contribution of *CLCN7* to lysosomal vacuolization in *FIG4* null cells. In *FIG4* null cells, there is reduction of the tonic down-regulation of the *CLCN7* transporter due to the reduced abundance of PI(3,5)P_2_; this results in excess influx of chloride ions and osmotic swelling. Knockout of the *CLCN7* chloride transporter eliminates the osmotic effect of excess inward flux of chloride ions. The data suggest that *CLCN7* is an important mediator of abnormal lysosomal dynamics in *FIG4* null and *VAC14* null cells. Many additional proteins contribute to the complex regulation of lysosomal pH and osmolarity[61,62].

The dominant negative mutant *CLCN7*-G215R causes defective trafficking of the ClC-7 transporter [42]. Heterozyzous mice carrying the corresponding G213R mutation exhibit mild osteopetrosis [15]. Combining G213R with the *FIG4* null mutation resulted in significant improvements in the mutant phenotype, with increased growth and muscle strength, and a 20% extension in lifespan from 38 days to 48 days. In recent work on the human *CLCN7* gain-of-function mutation Y715C, the enlarged vacuoles were corrected by overexpression of oculocutaneous albinism II (OCA2) Cl- ion channel, permitting exit of Cl^-^ and possibly of osmotically coupled water from the lysosome [49]. These observations support a connection between chloride overload in enlarged lysosomes and vacuole formation (Fig 10).

Mutations of *FIG4* and *CLCN7* both impair bone remodeling. In the *FIG4* null disorder Yunis-Varón Syndrome, osteoblast cultures accumulate large vacuoles and patients display multiple skeletal defects including reduced bone density, digital abnormalities and cleidocranial dysplasia [7], suggesting a defect in bone deposition. In contrast to bone loss in the *FIG4* null mutant, there is excessive bone matrix in patients with dominant negative mutations and reduced expression of *CLCN7* [27]. The G215R allele of *CLCN7*, used here to reduce *CLCN7* function in the mouse, was originally identified in a patient with dominant osteopetrosis [50] which results from impaired osteoclast-mediated bone resorption and is thus physiologically quite different from the deposition defect in FIG4 mutants. Hypopigmentation is another lysosome-related defect shared by loss-of-function of *FIG4* and gain-of-function of *CLCN7*, whose precise mechanism remains unclear [51].

Inhibitors of *CLCN7* have been investigated for treatment of osteoporosis [52,53]. These include micromolar levels of several non-specific inhibitors [42], blocking antibodies [54], and genetic down-regulation by antisense oligonucleotides and siRNA [55]. These strategies for reducing CLCN7 function are potential therapies for disorders caused by loss of function of *FIG4* and *VAC14*.

Our data implicate *CLCN7* in the osmotic swelling and hyperacidification of lysosomes in PI(3,5)P_2_ deficient cells caused by loss-of-function of *FIG4* or *VAC14*. Direct interaction between VAC14 and ClC-7 proteins was reported in the BioPlex Interactome 3.0 database [56], suggesting that the PI(3,5)P_2_ biosynthesis complex may be associated with ClC-7 in the lysosome membrane. Conversion of PI(3)P to PI(3,5)P_2_ may be a rate-limiting step in the reformation of lysosomes from autolysosomes [4,57–60]. The work described here identifies *CLCN7* as a potential target for treating neurodegenerative diseases caused by deficiency of PI(3,5)P_2_.

## Supporting information

S1 Fig A. Sequence of the 283 bp insert in exon 2 of the *FIG4* mutation generated in HAP1 null line F by Crispr/Cas9 targeting. Sanger sequencing of the amplified gene fragment. B. RTPCR product including the 283 bp insert. C. Sequence of the smaller product demonstrates use of an alternative splice acceptor site within the 283 bp insert.
(TIFF)

S2 Fig Protein truncation mutations of *CLCN7* in the two *FIG4*/*CLCN7* double null HAP1 cell lines. Genomic DNA was amplified by PCR. Products were separated by TA cloning and subjected to Sanger sequencing.
(TIFF)

S3 Fig pH calibration curve. HAP1 cells with WT genotype (black), *FIG4* null (pink), and double mutant FC7-1 (teal), were subjected to a ratiometric assay after setting the pH using pH-controlled bathing buffers and a combination of ionophores [21]. Each symbol represents the average lysosomal 490/435 fluorescence ratio from 14–15 cells, comprising 4–5 cells each from three independent experiments. The data are presented as mean ± standard deviation (SD).
(TIFF)

S4 Fig Staining of enlarged vacuoles in *FIG4* null cells by Oregon green and lysotracker. Representative live cell images of *FIG4* null HAP1 cells stained with OG488 (upper left) and Lysotracker blue (upper right). A merged image is shown at the lower left, and the transmitted light image at the lower right. Scale bar, 20 μm.
(TIFF)
