# Supplementary figures and images for: The chloride antiporter CLCN7 is a modifier of lysosome dysfunction in FIG4 and VAC14 mutants
Source: PLoS Genet. 2023 Jun 26;19(6):e1010800. doi: 10.1371/journal.pgen.1010800 (PMC10328317; doi:10.1371/journal.pgen.1010800)

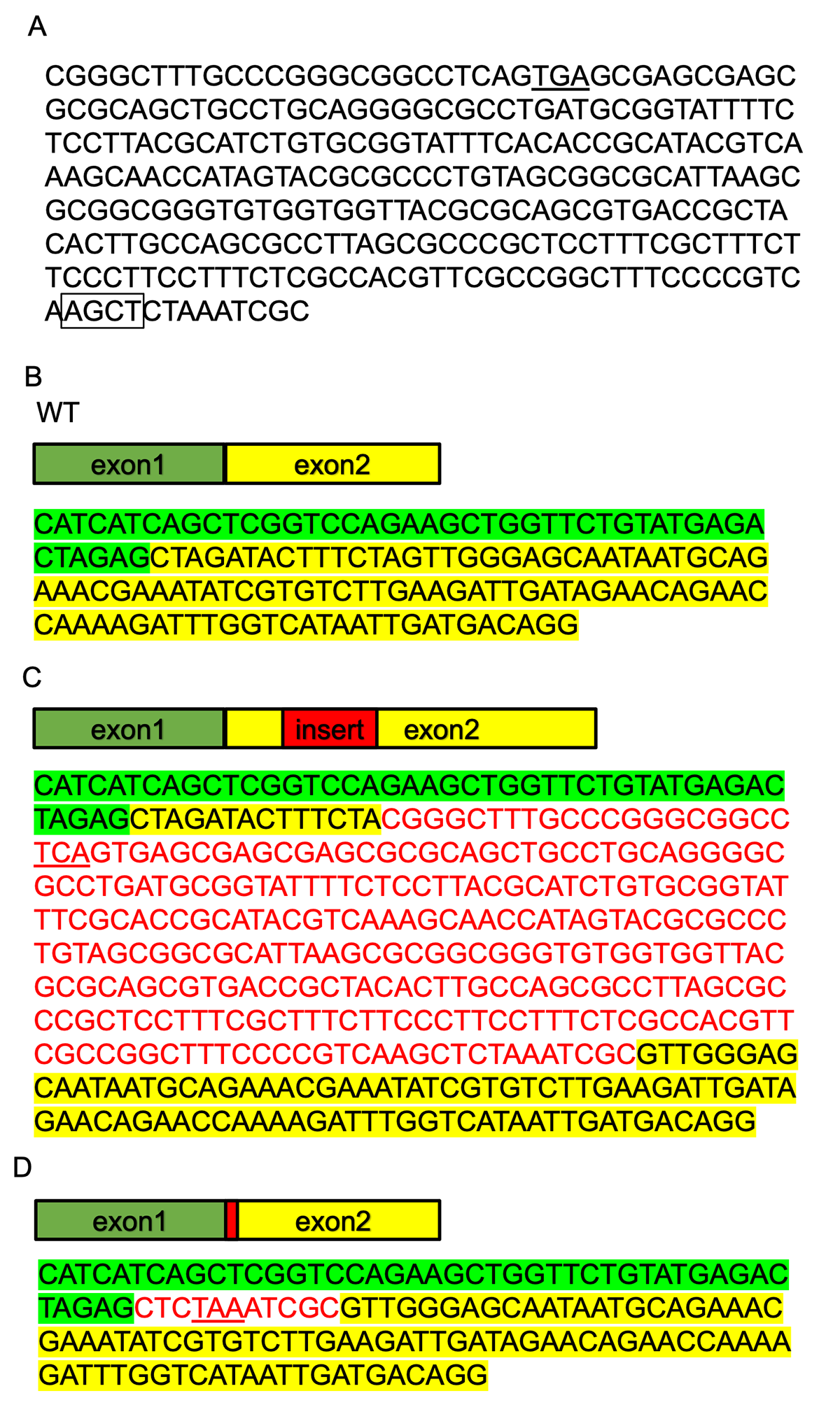

Supplement: S1 Fig — A. Sequence of the 283 bp insert in exon 2 of the FIG4 mutation generated in HAP1 null line F by Crispr/Cas9 targeting. Sanger sequencing of the amplified gene fragment. B. RTPCR product including the 283 bp insert. C. Sequence of the smaller product demonstrates use of an alternative splice acceptor site within the 283 bp insert. (TIFF) [file pgen.1010800.s001.tiff]

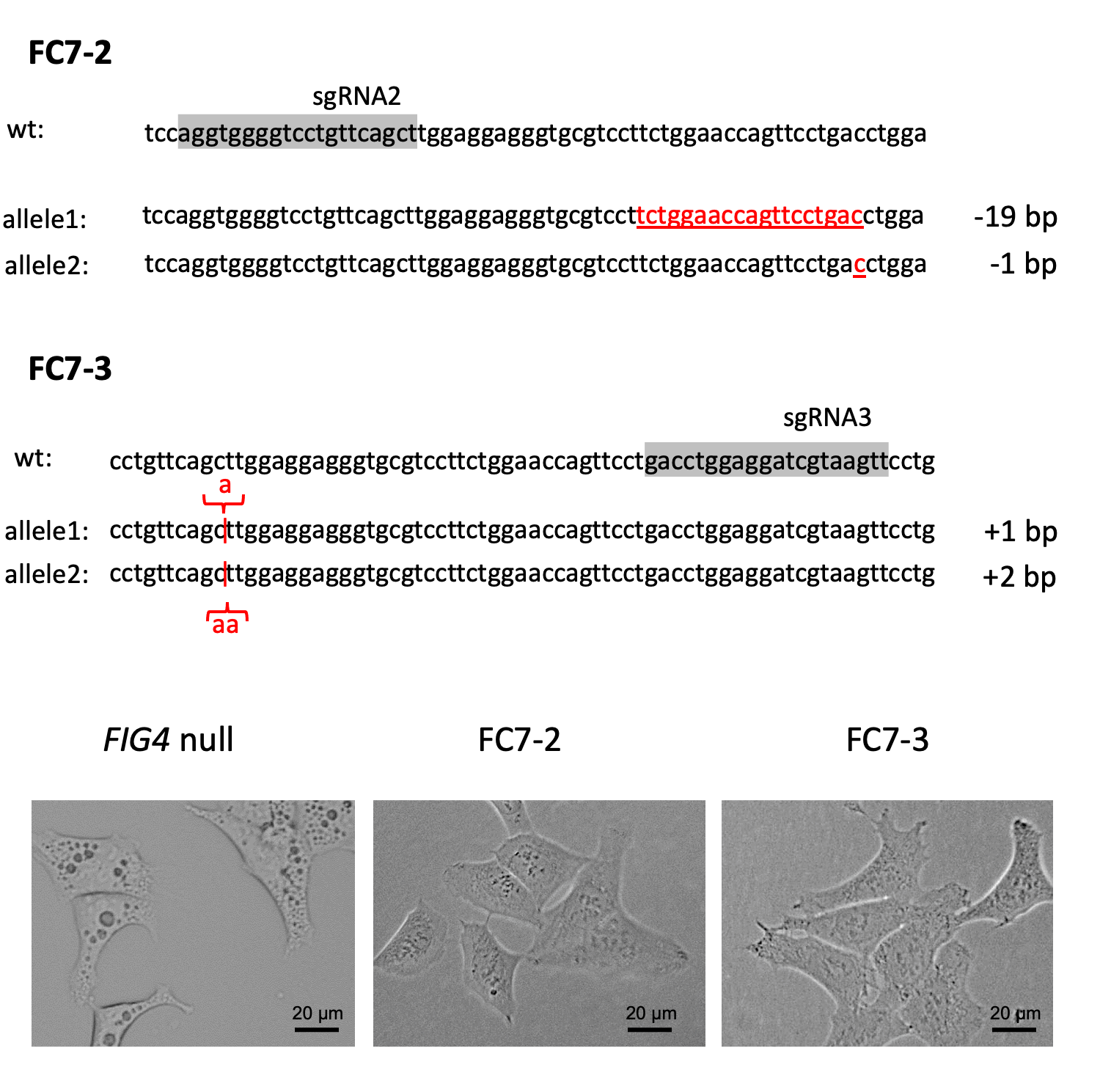

Supplement: S2 Fig — Genomic DNA was amplified by PCR. Products were separated by TA cloning and subjected to Sanger sequencing. (TIFF) [file pgen.1010800.s002.tiff]

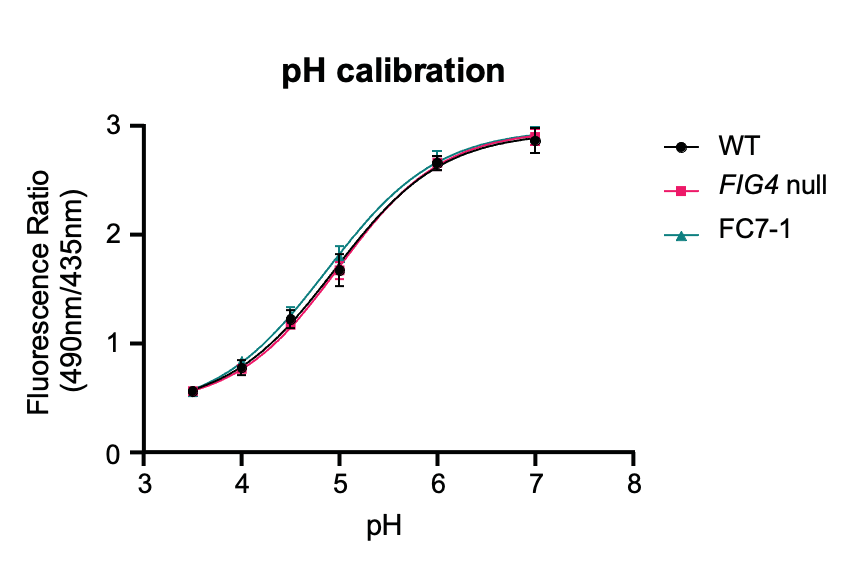

Supplement: S3 Fig — HAP1 cells with WT genotype (black), FIG4 null (pink), and double mutant FC7-1 (teal), were subjected to a ratiometric assay after setting the pH using pH-controlled bathing buffers and a combination of ionophores [21]. Each symbol represents the average lysosomal 490/435 fluorescence ratio from 14–15 cells, comprising 4–5 cells each from three independent experiments. The data are presented as mean ± standard deviation (SD). (TIFF) [file pgen.1010800.s003.tiff]

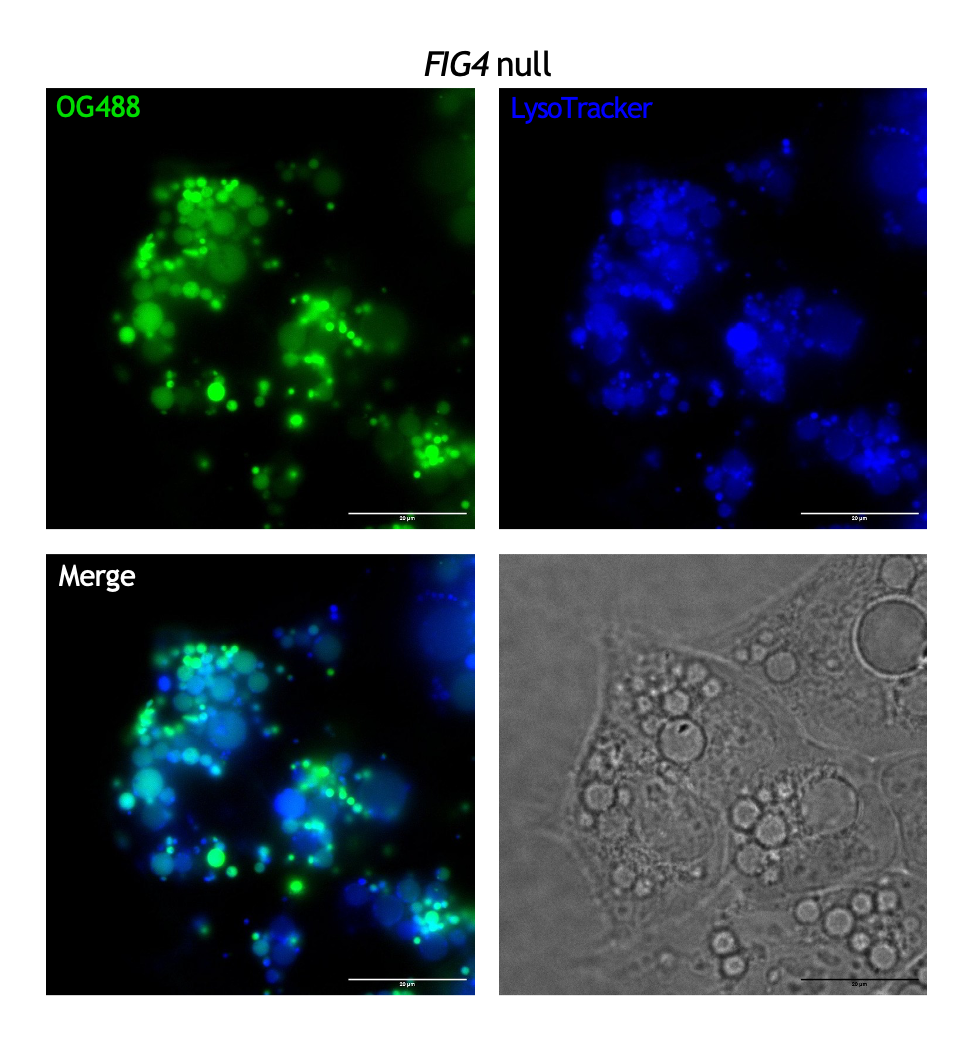

Supplement: S4 Fig — Representative live cell images of FIG4 null HAP1 cells stained with OG488 (upper left) and Lysotracker blue (upper right). A merged image is shown at the lower left, and the transmitted light image at the lower right. Scale bar, 20 μm. (TIFF) [file pgen.1010800.s004.tiff]
